# Effectiveness of motivational interviewing on medication adherence for the prevention of recurrent stroke or transient ischemic attack: Systematic review of randomized controlled trials

**DOI:** 10.1111/ene.16313

**Published:** 2024-04-27

**Authors:** Kathrin Wandscher, Falk Hoffmann, Christoph Heesen, Götz Thomalla, Anne Christin Rahn, Jasmin Helbach

**Affiliations:** ^1^ Department of Health Services Research, School of Medicine and Health Sciences Carl von Ossietzky University Oldenburg Oldenburg Germany; ^2^ Institute of Neuroimmunology and Multiple Sclerosis, Center for Molecular Neurobiology University Hospital Hamburg‐Eppendorf Hamburg Germany; ^3^ Department of Neurology University Hospital Hamburg‐Eppendorf Hamburg Germany; ^4^ Nursing Research Unit, Institute for Social Medicine and Epidemiology University of Lübeck Lübeck Germany

**Keywords:** medication adherence, motivational interviewing, stroke, systematic review, transient ischemic attack

## Abstract

**Background and purpose:**

This systematic review examines the effectiveness of motivational interviewing (MI) on medication adherence for preventing recurrent stroke and transient ischemic attack (TIA).

**Methods:**

MEDLINE (via PubMed), CINAHL, PsycINFO, CENTRAL, and ClinicalTrials.gov were searched from inception to 12 June 2023. Randomized controlled trials comparing MI with usual care or interventions without MI in participants with any stroke type were identified and summarized descriptively. Primary outcome was medication adherence. Secondary outcomes were quality of life (QoL) and different clinical outcomes. We assessed risk of bias with RoB 2 (revised Cochrane risk‐of‐bias tool) and intervention complexity with the iCAT_SR (intervention Complexity Assessment Tool for Systematic Reviews).

**Results:**

We screened 691 records for eligibility and included four studies published in five articles. The studies included a total of 2751 participants, and three were multicentric. Three studies had a high risk of bias, and interventions varied in complexity. Two studies found significantly improved medication adherence, one at 9 (96.9% vs. 88.2%, risk ratio = 1.098, 95% confidence interval = 1.03–1.17) and one at 12 months (97.0% vs. 95.0%, *p* = 0.026), but not at other time points, whereas two other studies reported no significant changes. No significant differences were found in QoL or clinical outcomes.

**Conclusions:**

Evidence on MI appears inconclusive for improving medication adherence for recurrent stroke and TIA prevention, with no benefits on QoL and clinical outcomes. There is a need for robustly designed studies and process evaluations of MI as a complex intervention for people with stroke.

**Registration:**

PROSPERO (CRD42023433284).

## INTRODUCTION

Stroke is one of the leading causes of death worldwide [1] and is associated with a high disease [2] and economic burden [3]. A stroke and a transient ischemic attack (TIA) are both caused by ischemia, differing only in the duration and severity of symptoms [4, 5], each with a high risk of recurrence [6]. The risk of a recurrent stroke increases from 11.1% in the first 12 months to 39.2% within the following 10 years [7].

Based on a current guideline [6], the risk of recurrent stroke or TIA can be reduced by an appropriate prevention strategy, including lifestyle changes and pharmacotherapy. However, people with stroke (PwS) show particularly low adherence to some of their prescribed medications (e.g., statins) [8], which might be due to polypharmacy, increased medication concerns, and low beliefs in their necessity [9]. Among behavioral approaches to address this specific issue, motivational interviewing (MI) appears to be promising [10, 11, 12, 13]. It is defined as “[…] a collaborative conversation style for strengthening a person's own motivation and commitment to change” [14]. MI facilitates behavior change through the following four sequential and recursive processes: (i) *Engaging*, which establishes a cooperative relationship between counsellor and client; (ii) *Focusing*, which helps identify and define goals with the client for guiding the next processes; (iii) *Evoking*, which assists clients in examining their motives and resolve any ambivalence toward change; and (iv) *Planning*, which involves creating a detailed action plan once the client commits to change. These processes are guided by the core skills of open‐ended questions, affirming, reflecting, and summarizing — practiced in the underlying spirit of partnership, evocation, acceptance, and compassion [14].

Previous reviews of randomized controlled trials (RCTs) have largely indicated improvements in medication adherence following MI interventions in people with chronic conditions [10, 11, 12], including cardiovascular diseases (CVDs) [13]. Especially in CVDs, MI appears to achieve a higher medication adherence compared to other chronic conditions [11]. However, the effects of MI seem to vary across the different CVDs [11, 13] and have not yet been systematically investigated separately for stroke or TIA.

Therefore, this systematic review aims to examine the effectiveness of MI on medication adherence for stroke and TIA recurrence prevention.

## METHODS

We conducted this systematic review following the methodological framework described by the Cochrane Handbook for Systematic Reviews of Interventions [15]. We reported according to the Preferred Reporting Items for PRISMA (Systematic Reviews and Meta‐Analyses) statement [16]. The protocol was registered in PROSPERO (CRD42023433284).

### Eligibility criteria

We included published individual or cluster RCTs using parallel group designs (including multiarm trials) with a sample size of ≥50 participants. Other study types, dissertations, and conference abstracts were excluded. There were no restrictions on study length, date, or publication language.

Eligibility criteria were defined using the PICO (population, interventions, comparators, outcomes) approach for reviews assessing the effectiveness of interventions [17].

### Population

We included studies of participants with any type of stroke, both ischemic and hemorrhagic (including intracranial or subarachnoid hemorrhage), or TIA within the past year, irrespective of gender or level of poststroke disability. Studies including participants with prescribed preventive stroke medication, defined as any antiplatelet, anticoagulant, antihypertensive, or lipid‐lowering agent [6], were eligible. We excluded studies whose results were reported solely for hemorrhagic stroke due to trauma and studies including only children. The study setting was not restricted.

### Intervention/comparator

We included interventions labeled as MI, either as a single intervention or as part of a combined intervention, addressing medication adherence alone or in combination with lifestyle changes. MI could be delivered face‐to‐face, by telephone, or combined by trained counselors regardless of professional background, session number, or length. As a comparator, usual care and any other intervention without MI or wait‐list control groups compared to MI interventions were included. We excluded group interventions or those not delivered by a person.

### Outcome

The primary outcome was adherence to preventive medications as measured by the study authors' criteria. Secondary outcomes were quality of life (QoL), stroke or TIA recurrence, cardiovascular hospitalization, all‐cause death, or a composite of 3‐point major adverse cardiovascular events (3P‐MACE), including cardiovascular death, myocardial infarction, and stroke.

### Data sources and search

We searched the electronic databases MEDLINE (via PubMed), CINAHL (via EBSCOhost), PsycINFO (via EBSCOhost), and CENTRAL (via Cochrane Library), and the trial register ClinicalTrials.gov (https://clinicaltrials.gov/) from inception to 12 June 2023. We adapted the search strategies for stroke/TIA [18] and MI [12] from previous reviews and combined them with a modified version of the RCT filters developed by the Cochrane Collaboration [19]. For searching ClinicalTrials.gov, keywords from a previous review [20] were used (Table S1). Additionally, we manually screened the reference lists of included articles and conducted a forward‐citation search of these articles using Web of Science on 9 July 2023.

### Study selection and data extraction

We imported the search results into an EndNote library (Version 20.5, Clarivate, Philadelphia, PA, USA) and then to Rayyan [21] after removing all duplicates. All titles/abstracts were independently screened for inclusion or exclusion by two reviewers (J.H., K.W.). The screening process was piloted on 50 titles/abstracts to calibrate reviewers. The same reviewers independently assessed the full texts of all articles, meeting the inclusion criteria. Discrepancies were resolved by discussion or by a third reviewer (F.H.).

We extracted the following data: authors, publication year, study characteristics (e.g., study design, country), participant characteristics (e.g., age, gender, stroke types), eligibility criteria, intervention description, comparator description, and outcome information (e.g., measures, results). If studies reported multiple results, the following were preferred: continuous outcomes (when reporting means, standard deviations, sample sizes), unadjusted effect estimates, confidence intervals (CIs), and intention‐to‐treat analysis. We obtained further information from available supplementary material or study protocols. One reviewer (K.W.) extracted data directly into the results tables, which were verified by a second reviewer (J.H.). Discrepancies were resolved by discussion or by a third reviewer (F.H.).

### Intervention complexity assessment

One reviewer (K.W.) assessed the intervention complexity of the included articles using the intervention Complexity Assessment Tool for Systematic Reviews (iCAT_SR) [22] for six core and four optional dimensions, which were verified by a second reviewer (J.H.). The intervention complexity was rated according to criteria and levels provided by the iCAT_SR guidance [22] and then visualized based on the categories of a previous study [23]. Discrepancies were resolved by discussion or by a third reviewer (F.H.).

### Risk of bias assessment

Two reviewers (J.H., K.W.) independently assessed the risk of bias of included studies using the revised Cochrane risk‐of‐bias (RoB 2) tool [24] for the primary outcome of this review. We rated the risk of bias according to the algorithm and criteria provided by RoB 2 for the following domains: randomization process, deviations from intended interventions (assignment to the intervention), missing outcome data, measurement of the outcome, selection of the reported result, and overall risk of bias [24]. Given the small number of included studies, we conducted a calibration exercise using three studies from a review on MI for coronary heart disease [11]. Discrepancies were resolved by discussion or by a third reviewer (F.H.).

### Data synthesis

The data were summarized descriptively (narrative synthesis). Given the expected heterogeneity in measuring medication adherence between studies [11, 13], a meta‐analysis was not planned. We linked multiple included articles of the same study and reported them at the study level.

## RESULTS

### Study selection

**FIGURE 1:**
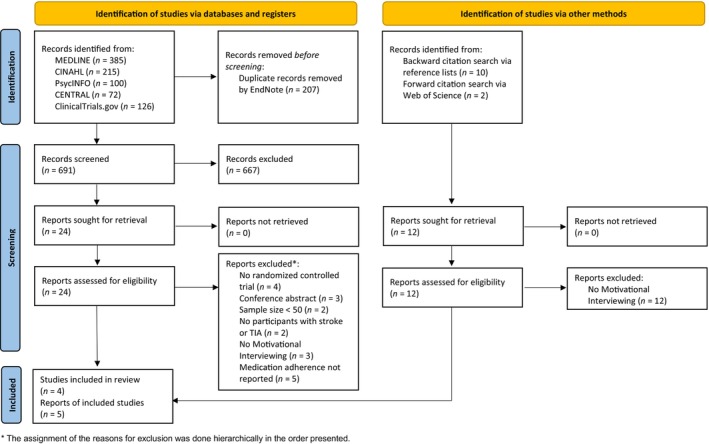
PRISMA (Systematic Reviews and Meta‐Analyses) flow diagram. CINAHL, Cumulative Index to Nursing and Allied Health Literature; TIA, transient ischemic attack.

The searches identified 691 records (Figure 1). After title/abstract screening, 24 full texts were read, and five articles reporting on four studies were included [25, 26, 27, 28, 29]. All articles were written in English. Table S2 shows the excluded articles with reasons for exclusion.

### Study and participant characteristics

The characteristics of all included studies are presented in Table 1. All articles were individual, two‐arm RCTs published between 2013 and 2022 [25, 26, 27, 28, 29]. One study each was conducted in Canada [29], Denmark [28], and New Zealand [27], and one was conducted in Denmark and Germany [25, 26]. The setting was inpatient (*n* = 2 studies) [27, 28], outpatient (*n* = 1) [29], or both (*n* = 1) [25, 26], and most studies were multicentric (*n* = 3) [25, 26, 27, 29]. The study duration ranged from 18 to 62 months [25, 26, 28, 29].

**TABLE 1:** Characteristics of the included studies.

Authors (year)	Study design; study duration	Country	Number of randomized participants; setting	Inclusion and exclusion criteria	Type of stroke (%)	Concomitant medication, %	Comorbidities, %	Level of disability	Age of included participants, years; % female
Ahmadi et al. [25] (2020) with companion report: Ihl et al. [26] (2022)^a^	Two‐arm parallel, multicenter RCT; August 2011 to October 2017	Germany and Denmark	*n* = 2098; 7 German hospitals and 1 Danish stroke center	**Inclusion:** Ischemic stroke, intracerebral hemorrhage, TIA, or ischemic monocular blindness within past 14 days, aged ≥18 years, at least 1 modifiable risk factor, nondisabling deficits (mRS score ≤ 2), living within 20 miles of intervention facilities **Exclusion:** ABCD score ≤ 2 and no acute ischemic lesion on cerebral imaging, stroke, or TIA with atypical causes like dissection or vasculitis, malignant disease with life expectancy < 3 years, severe cognitive impairment, current substance dependency	IG: Ischemic stroke (59.5%), ICH (1.1%), TIA (37.4%), ischemic monocular blindness (2.0%) CG: Ischemic stroke (58.8%), ICH (0.7%), TIA (39.0%), ischemic monocular blindness (1.5%)	n.r.	IG: HTN (86.7%), DM (23.8%), AF (16.6%) CG: HTN (88.8%), DM (24.3%), AF (17.1%)	IG: MoCA = 25.1 (mean); mRS = 1 (median) CG: MoCA = 25.1 (mean), mRS = 1 (median)	IG: 67.1 (mean); 34.2% female CG: 67.7 (mean); 33.3% female
Barker‐Collo et al. [27] (2015)	Two‐arm parallel, multicenter RCT^b^; study period: n.r.	New Zealand^b^	*n* = 386; 4 hospitals^b^	**Inclusion:** First‐time stroke within past 28 days defined according to the WHO criteria and confirmed by a diagnostic review committee, aged ≥16 years, residents of 2 regions nearby the intervention facilities^b^ **Exclusion**: Diagnosis of SAH, discharged to hospital or nursing home, impairments precluding participation, another condition likely to impact their participation, undergoing psychiatric/psychological treatment, participation was likely to overburden, unable to speak in English, inability to give informed consent	n.r.	IG: ASA (39.9%), dipyridamole (4.1%), clopidogrel (3.6%), vitamin‐k‐antagonist (4.1%), statins (31.6%), antihypertensives (56.0%) CG: ASA (37.2%), dipyridamole (2.1%), clopidogrel (3.1%), vitamin‐k antagonist (4.1%), statins (35.8%), antihypertensives (54.9%)	n.r.	n.r.	n.r.
Hedegaard et al. [28] (2014)	Two‐arm parallel, single‐center RCT; August 2012 to March 2014	Denmark	*n* = 211; 1 hospital	**Inclusion:** First‐time ischemic stroke or TIA within past 30 days, aged ≥18 years, prescribed ≥1 antiplatelet or anticoagulant agent, medication dispensed by themselves or cohabiting relatives **Exclusion:** Living in a nursing home or institution, receiving pharmacy or home nurse dispensed medications, terminal illness, cognitive or physical impairment	IG: Ischemic stroke (52.0%), TIA (46.1%) CG: Ischemic stroke (49.5%), TIA (48.5%)	IG: Antiplatelets (94.1%), ASA and dipyridamole (67.6%), clopidogrel (22.5%), oral anticoagulants (5.9%), statins (80.4%), antihypertensives (54.9%) CG: Antiplatelets (96.0%), ASA and dipyridamole (72.3%), clopidogrel (19.8%), oral anticoagulants (5.0%), statins (74.3%), antihypertensives (49.5%)	IG: HTN (53.9%), AF (6.9%), DM (7.8%), DLD (83.3%) CG: HTN (45.5%), AF (9.9%), DM (6.9%), DLD (75.2%)	n.r.	IG: 64 (median); 40.1% female CG: 68 (median); 37.6% female
Mackenzie et al. [29] (2013)	Two‐arm parallel, multicenter RCT; April 2010 to October 2011	Canada	*n* = 56; 4 nurse‐led secondary stroke prevention clinics	**Inclusion**: Confirmed stroke or probable TIA diagnosed by a stroke prevention clinic physician, aged ≥18 years, uncontrolled HTN, MoCA < 26, medication self‐efficacy < 100%, self‐report of missed pills **Exclusion:** Living in a retirement or nursing facility, receiving caregiver dispensed medications, unable to speak or read in English, inability to give informed consent	Stroke (64.3%) and TIA (35.7%) combined for both IG and CG	n.r.	HTN (100%), HCL (73.2%), DM (32.1%), and AF (3.6%) combined for both IG and CG	MoCA = 23.3 (mean) combined for both IG and CG	58.9% age > 65 years (age group) combined for both IG and CG; 32.1% female combined for both IG and CG

Abbreviations: ABCD, age, blood pressure, clinical features, duration of symptoms; AF, atrial fibrillation; ASA, acetylsalicylic acid; CG, control group; DLD, dyslipidemia; DM, diabetes mellitus; HCL, hypercholesterolemia; HTN, hypertension; ICH, intracerebral hemorrhage; IG, intervention group; MoCA, Montreal Cognitive Assessment; mRS, modified Rankin Scale; n.r., not reported; RCT, randomized controlled trial; SAH, subarachnoid hemorrhage; TIA, transient ischemic attack; WHO, World Health Organization.

^a^ Data obtained only from the report by Ahmadi et al. [25].

^b^ Data obtained from the referred study protocol by Krishnamurthi et al. [31].

The four studies included a total of 2751 participants (intervention: *n* = 1374; control: *n* = 1377) with a mean/median age ranging from 64 to 68 years (*n* = 2) [25, 26, 28] and a proportion of females from 32.1% to 40.1% (*n* = 3) [25, 26, 28, 29]. Study enrollment after a diagnosed stroke or TIA event ranged from 14 to 30 days (*n* = 3) [25, 26, 27, 28], which were only first‐time events in two studies [27, 28]. The stroke type ranged from 35.7% to 48.5% for TIA (*n* = 3) [25, 26, 28, 29] and 49.5% to 59.5% for ischemic stroke (*n* = 2) [25, 26, 28]. In one study, the stroke type was unclear [27]. Two studies included participants with mild cognitive impairments [25, 26, 29] and one with medication nonadherence [29]. Three studies reported comorbidities [25, 26, 28, 29] and two preexisting medications [27, 28].

### Intervention description

Across the four studies, the MI interventions were heterogenous (Table 2). In all studies, the MI interventions were delivered by different professionals with varying levels of training. The types of delivery included either face‐to‐face interviews (*n* = 1) [25, 26], telephone interviews (*n* = 1) [29], or both (*n* = 2) [27, 28]. Regarding behavior change, MI was combined with two or more intervention components (*n* = 3) [25, 26, 28, 29], addressing only medication adherence (*n* = 1) [28] or also lifestyle changes (*n* = 3) [25, 26, 27, 29], by applying only one (*n* = 2) [25, 26, 28] or all processes (*n* = 1) [27] of MI. The interventions lasted from 6 to 24 months (*n* = 4) [25, 26, 27, 28, 29], with a total session number ranging from four to eight (*n* = 3) [25, 26, 27, 28], each lasting from 15 to 90 min (*n* = 2) [27, 28].

**TABLE 2:** Overview of the interventions of the included studies.

Characteristics of the intervention	Authors (year)			
	Ahmadi et al. [25] (2020) with companion report: Ihl et al. [26] (2022)^a^	Barker‐Collo et al. [27] (2015)	Hedegaard et al. [28] (2014)	Mackenzie et al. [29] (2013)
Intervention provider	Physicians^b^	Researchers	Pharmacists	Nursing specialists
MI training	2 days with 1‐day refresher course after 6 months	Ongoing training throughout the study^c^	3 days with 1‐day refresher course after 1 month	Prior education
Type of delivery	Face‐to‐face interviews	Initial face‐to‐face interview with subsequent face‐to‐face or telephone interviews	Initial face‐to‐face interview with subsequent telephone interviews	Telephone interviews
Intervention components	MI, risk factors assessment, education, additional resources, written recommendations	MI	MI, medication review, medication problems,^d^ written session summary	MI, home BP monitoring, medication dosettes
Content of MI (behavioral targets)	Plan for risk factor modification based on risk factor assessment (medication adherence and lifestyle changes^e^)	Establish a relationship, focus on identifying and defining goals, motivation toward change, plan for risk factor modification (medication adherence and lifestyle changes^d^)	Focus on identifying and defining goals based on medication review (medication adherence)	n.r. (medication adherence and lifestyle changes)
Sessions, *n* (session duration, min)	8 (n.r.)	4 (30–90)	4 (15–30)	n.r. (n.r.)
Intervention duration (time points)	24 months (3 and 6 weeks, and 3, 6, 9, 12, 18, and 24 months)	9 months (1, 3, 6, and 9 months)	6 months (baseline, 1 week, and 6 months)	6 months (at least monthly)

Abbreviations: BP, blood pressure; MI, motivational Interviewing; n.r., not reported.

^a^ Data were obtained from the report of Ahmadi et al. [25] unless otherwise indicated.

^b^ MI training for nurses was reported only by Ahmadi et al. [25] not by Ihl et al. [26] or the study protocol by Leistner et al. [30].

^c^ Data obtained from the referred study protocol by Krishnamurthi et al. [31].

^d^ Additional data from supplemental material.

^e^ Data obtained from the referred study protocol by Leistner et al. [30].

A detailed description of the interventions is presented in Table S3. For a standardized approach, two studies used interview guidelines [27, 28] and one MI scripts [29]. Two studies allowed relatives to participate during the sessions [25, 26, 28] and one adaptions for the type of delivery [27]. MI was used for all sessions in three studies [25, 26, 27, 29], and in the other study, MI was required only in the first session [28]. Two studies used an intervention component preceding MI, either a risk factor assessment [25, 26] or a medication review [28]. All studies included usual care for the control groups [25, 26, 27, 28, 29].

### Intervention complexity

Regarding the 10 iCAT_SR dimensions, the intervention complexity was high in all four studies for “dependency of intervention effects by individuals” and in at least half of the studies across four dimensions (Figure S1). All four studies were unclear or not assessable for two dimensions (Table S4).

### Risk of bias

In total, three of four studies had a high overall risk of bias, and the remaining study raised some concerns. Of the five remaining bias domains, three studies had a high risk of bias for “outcome measurement” and one for “missing outcome data” (Figure S2 and Table S5).

### Primary outcome: Medication adherence

All four studies assessed medication adherence using different measures (Table 3). Whereas Ahmadi et al. [25] and Barker‐Collo et al. [27] reported at least one significant change between the intervention group (IG) and control group (CG) in adherence to antiplatelet agents or in overall adherence, the remaining two studies found no significant differences [28, 29].

**TABLE 3:** Outcomes and results for medication adherence of the included studies.

Authors (year)	Primary outcome: medication adherence	Measurement	Adherence cutoff point	Effect on medication adherence	Analysis method
Ahmadi et al. [25] (2020) with companion report: Ihl et al. [26] (2022)^a^	Adherence to antiplatelets or oral anticoagulants	Antiplatelets: Standardized written questionnaire (measure not specified)^b^ Oral anticoagulants: Blood samples collected by trained study nurses	Antiplatelets: n.r. Oral anticoagulants: INR = 2–3	12 months: Antiplatelets: IG (*n* = 621): 97.0% vs. CG (*n* = 643): 95.0%, *p* = 0.026 Oral anticoagulation: IG (*n* = 172): 90.0% vs. CG (*n* = 174): 86.0%, *p* = 0.34 Oral anticoagulation on target (INR = 2–3): IG (*n* = 172): 83.0% vs. CG (*n* = 174): 75.0%, *p* = 0.055 24 months: Antiplatelets: IG (*n* = 468): 98.0% vs. CG (*n* = 467): 96.0%, *p* = 0.12 Oral anticoagulation: IG (*n* = 146): 90.0% vs. CG (*n* = 122): 89.0%, *p* = 0.62 Oral anticoagulation on target (INR = 2–3): IG (*n* = 146): 82.0% vs. CG (*n* = 122): 78.0%, *p* = 0.38 36 months: Antiplatelets: IG (*n* = 382): 96.0% vs. CG (*n* = 358): 95.0%, *p* = 0.71 Oral anticoagulation: IG (*n* = 119): 88.0% vs. CG (*n* = 108): 90.0%, *p* = 0.70 Oral anticoagulation on target (INR = 2–3): IG (*n* = 119): 81.0% vs. CG (*n* = 108): 79.0%, *p* = 0.71	ITT + PP
Barker‐Collo et al. [27] (2015)	Self‐reported adherence	Closed‐ended question about taking medication as prescribed in the past 7 days and number of missed doses with reasons for each drug, which was cross‐checked with available electronic medication dispensation records	n.r.	3 months: IG (*n* = 172): 91.9% vs. CG (*n* = 176): 90.9%, RR = 1.011 (95% CI = 0.95 to 1.08) 6 months: IG (*n* = 175): 92.0% vs. CG (*n* = 171): 85.4%, RR = 1.078 (95% CI = 1.00 to 1.16) 9 months: IG (*n* = 159): 96.9% vs. CG (*n* = 161): 88.2%, RR = 1.098 (95% CI = 1.03 to 1.17) 12 months: IG (*n* = 161): 93.2% vs. CG (*n* = 165): 92.7%, RR = 1.005 (95% CI = 0.95 to 1.07)	ITT
Hedegaard et al. [28] (2014)	Overall adherence: Preventive medication including antiplatelets, oral anticoagulants, and statins Specific adherence: ASA, dipyridamole, clopidogrel, statins, and antihypertensives Persistence: Clopidogrel, ASA, dipyridamole, and statins	*Overall adherence:* MPR based on the OPED Specific Adherence: MPR based on the OPED Persistence: Based on the OPED	Nonadherence: MPR < 0.80 Persistence: Redeem a prescription within 90 days of the previous prescription	Baseline: Overall: Nonadherent: IG (*n* = 59): 40.7% vs. CG (*n* = 48): 52.1%, (CI or *p*‐value not reported) 3, 6, and 9 months: The authors indicated that there were no significant differences in overall adherence when comparing groups^c^ 12 months: Overall: Nonadherent: IG: 28.0% vs. CG: 21.0%,^d^ RD = 7.0% (95% CI = −15.0% to 19.0%) ASA: Nonadherent: IG: 26.0% vs. CG: 28.0%,^d^ RD = −2.0% (95% CI = −15.0% to 12.0%) Nonpersistence: IG (*n* = 56): 27.0% vs. CG (*n* = 67): 36.0%, HR = 0.74 (95% CI = 0.37 to 1.40) Dipyridamole: Nonadherent: IG: 25.0% vs. CG: 22.0%,^d^ RD = 4.0% (95% CI = −10.0% to 18.0%) Nonpersistence: IG (*n* = 48): 10.0% vs. CG (*n* = 57): 11.0%, HR = 1.02 (95% CI = 0.31 to 3.33) Clopidogrel: Nonadherent: IG: 13.0% vs. CG: 5.0%,^d^ RD = 8.0% (95% CI = −5.0% to 20.0%) Nonpersistence: IG (*n* = 21): 10.0% vs. CG (*n* = 19): 11.0%, HR = 0.83 (95% CI = 0.12 to 5.92) Statins: Nonadherent: IG: 22.0% vs. CG: 21.0%,^d^ RD = 2.0% (95% CI = −11.0% to 14.0%) Nonpersistence: IG (*n* = 74): 20.0% vs. CG (*n* = 67): 18.0%, HR = 1.23 (95% CI = 0.57 to 2.62) Antihypertensive agents: Nonadherent: IG: 22.0% vs. CG: 25.0%,^d^ RD = −3.0% (95% CI = −18.0% to 11.0%)	PP
Mackenzie et al. [29] (2013)	Missed pills: Self‐reported adherence Pharmacist review: Community pharmacist review of participant prescription renewal patterns for 1 to 3 medications	Missed pills: Open‐ended question about the number of missed pills in an average week with reasons Pharmacist review: Closed‐ended question about participants adhering to prescription refills 80% or more of the time	n.r.	Baseline: Missed pills: IG (*n* = 29): 0.5 ± 0.9 vs. CG (*n* = 27): 0.7 ± 1.5, *p* = 0.58 Pharmacist review: IG (*n* = 29): 80.5% ± 35.0% vs. CG (*n* = 27): 119.3% ± 190.4%, *p* = 0.33 6 months: Missed pills: IG (*n* = 29): 34.5 ± 185.5 vs. CG (*n* = 27): 37.6 ± 192.1, *p* = 0.95 Pharmacist review: IG (*n* = 29): 125.6% ± 188.4% vs. CG (*n* = 27): 83.3% ± 34.1%, *p* = 0.28	n.r.

Abbreviations: ±, standard deviation; ASA, acetylsalicylic acid; CG, control group; CI, confidence interval; HR, hazard ratio; IG, intervention group; INR, international normalized ratio; ITT, intention‐to‐treat; MPR, medication possession ratio; n.r., not reported; OPED, Odense Pharmacoepidemiological Database; PP, per‐protocol; RD, risk difference; RR, risk ratio.

^a^ Medication adherence measure and its results were only obtained from the report of Ahmadi et al. [25].

^b^ Data obtained from the referred study protocol by Leistner et al. [30].

^c^ No data reported in text, and data reported visually could not be estimated due to missing denominator separately for IG and CG.

^d^ Denominator was not reported.

Ahmadi et al. [25] reported significant changes in adherence to antiplatelet agents at 12 months (IG [*n* = 621]: 97.0% vs. CG [*n* = 643]: 95.0%, *p* = 0.026), with no significant differences at 24‐ and 36‐month follow‐up, and no significant changes in adherence to oral anticoagulants (only intake or within the therapeutic target range of the international normalized ratio [INR]) during follow‐up. Barker‐Collo et al. [27] reported significant differences in overall adherence at 9 months (IG [*n* = 159]: 96.9% vs. CG [*n* = 161]: 88.2%, risk ratio [RR] = 1.098, 95% CI = 1.03–1.17), with no significant changes at 3‐, 6‐, and 12‐month follow‐up. In Hedegaard et al. [28], there were no significant differences in overall adherence to preventive medications (including antiplatelet agents, oral anticoagulants, and statins) at 3‐, 6‐, 9‐, and 12‐month follow‐ups, in adherence to statins, antihypertensives, or single antiplatelet agents at 12 months, and persistence on statins or single antiplatelet agents at 12 months. Mackenzie et al. [29] reported no significant differences for missed pills and pharmacist reviews of prescription renewals at 6 months.

### Secondary outcomes: QoL and clinical endpoints

Two studies assessed QoL using different instruments [26, 27]. Ihl et al. [26] reported no significant differences for the EuroQoL five‐dimensional three‐level instrument at follow‐up. Barker‐Collo et al. [27] found no significant changes for all subscales of the 36‐item Short Form Health Survey during follow‐up.

All four studies assessed at least one clinical endpoint, with three using different and multiple data sources and one not specifying (Table S6). Three studies reported no significant differences in stroke recurrence at follow‐up [25, 27, 29]. Regarding TIA recurrence, Barker‐Collo et al. [27] detected no significant changes at 12 months (IG [*n* = 193]: 2.1% vs. CG [*n* = 193]: 2.1%, RR = 1.00, 95% CI = 0.25–3.94). Ahmadi et al. [25] found no significant differences in all‐cause deaths (IG [*n* = 1030]: 7.1% vs. CG [*n* = 1042]: 8.2%, hazard ratio = 0.85, 95% Cl = 0.62–1.17), major vascular hospitalizations (IG: 330/1000 patient‐years [PY] vs. CG: 359/1000 PY, incidence rate ratio [IRR] = 0.92, 95% Cl = 0.79–1.07), and other vascular hospitalizations (IG: 158/1000 PY vs. CG: 164/1000 PY, IRR = 0.97, 95% Cl = 0.78–1.20) at 60 months. Two studies reported no significant changes for 3P‐MACE at follow‐up [25, 28].

## DISCUSSION

In this systematic review, we found that MI has inconclusive effects on medication adherence for stroke and TIA recurrence prevention. Positive effects of MI on medication adherence were reported by two studies, each only for a single time point [25, 27], whereas the others found no effects overall [28, 29]. No studies showed positive effects on QoL or any clinical endpoint [25, 26, 27, 28, 29]. Furthermore, we found that MI interventions were complex and heterogeneous.

Currently, there is no other systematic review of RCTs assessing the effectiveness of MI for medication adherence in PwS. Nevertheless, our findings of small to no improvements of MI on medication adherence are in contrast to previous reviews showing largely beneficial effects in populations with chronic conditions [10, 11, 12] and particularly in CVDs [11]. These conflicting results might partly be explained by stroke‐related factors at study inclusion, such as cognitive impairments [14], first‐time or recurrent events [28], or the start of the intervention shortly after a stroke or TIA. Additionally, most of the included PwS had high medication adherence levels of 80% or more in both the IG and CG [25, 27, 28], which might indicate ceiling effects. That this high adherence level does not mirror the overall evidence is demonstrated in a previous review showing that the overall level of medication adherence was only approximately 64% in PwS [8]. Thus, these high levels might leave less room for improvements [28]. However, less is known about the influence of the overall adherence levels or condition‐related factors on the effectiveness of MI for medication adherence [11, 12] in PwS. Another explanation could be the use of nonvalidated measures to assess medication adherence in most studies [25, 27, 29, 30, 31]. Interestingly, two of these studies reported significantly improved medication adherence at 9 [27] and 12 months [25]. However, whether these small improvements [25, 27] could be considered clinically relevant is debatable. Furthermore, most of the included studies had brief follow‐up periods [27, 28, 29], which might be too short to detect relevant long‐term effects.

The included studies found no beneficial effects for QoL [26, 27] or any clinical endpoint [25, 27, 28, 29]. An explanation might be that the little to no improvements in medication adherence [25, 27, 28, 29] could not lead to better clinical outcomes [12] or QoL. In addition, QoL was only assessed in two studies using different instruments [26, 27], and clinical outcomes were rarely considered in most studies except for stroke recurrence [25, 27, 28, 29]. However, there is a general lack of evidence on the effects of MI on clinical outcomes [10, 12] and QoL in PwS, and therefore no conclusions could be drawn. Otherwise, the results for clinical outcomes might be more likely explained by the short follow‐up periods in three studies [27, 28, 29]. Furthermore, all assessed outcomes were measured as secondary outcomes in most of the included studies [27, 28, 29], thus the sample sizes might be too small to detect important rare events.

Across the included studies, the MI interventions were largely different [25, 27, 28, 29]. This is in line with previous reviews demonstrating the same large heterogeneity regarding the number and extent of the intervention components, providers [11], delivery modes [10], MI training [10, 13], and MI processes used. Even though MI is an evidence‐based approach that can be combined with different intervention components and adapted to various contexts [14], it is difficult to distinguish the influence of each single component [10, 11, 12]. This is further hampered by our iCAT_SR results showing a high intervention complexity regarding the number of components and their interactions, as well as the range of targeted behaviors in half of the studies. Moreover, the iCAT_SR results from most studies indicate that intervention providers need to be highly skilled, and thus fidelity in the delivery of MI might be an important factor, as its effects can vary across providers and settings within a study [14]. Overall, the iCAT_SR results indicate that MI interventions appear to be complex and address most key characteristics of complex interventions as defined by the Medical Research Council (MRC) framework [32].

For complex interventions, the MRC framework emphasizes developing a program theory based on existing evidence and theories, outlining their main components, and describing how they are intended to work in a given context [32]. However, our iCAT_SR results show that none of the included studies described how the MI interventions might improve medication adherence, QoL, and clinical endpoints or considered their interaction within the context. Moreover, the MRC framework implies also the need of process evaluations to better understand why a complex intervention works or does not work and how it could be optimized [32]. However, there is a lack of evidence on how different elements of MI interventions influence the effectiveness of different outcomes in people with chronic diseases [10, 11, 12] or stroke. Therefore, future studies could benefit from developed program theories of MI interventions and process evaluations for an understanding of how they lead or do not lead to improved results within the given context.

The review's main strength was its comprehensive search strategy without restrictions regarding language or publication period of articles. Nevertheless, there is a possibility that we may have missed studies reporting on MI or medication adherence. To minimize this risk, we screened full texts of all RCTs reporting on psychological interventions for PwS and conducted a forward and backward citation search of all included articles. However, the interventions may not have been labeled as MI. Moreover, the comparability of the included studies is limited by their heterogeneity regarding participants, interventions, intervention providers, medication adherence measures, and the different health care systems across countries. Additionally, our findings require cautious interpretation because of the overall high risk of bias in three of four studies, highlighting potential problems with outcome measures in open‐label trials. The study quality was assessed using the RoB 2 tool [24], and the results were reported transparently with reasons for our judgments. Despite conducting a calibration exercise for RoB 2, our judgments of “measurement of outcome” and “selection of reported results” for two included studies [27, 28] differed from those of a previous review [11]. However, applying the RoB 2 tool is challenging and demanding, even for assessors with extensive experience in conducting systematic reviews and assessing risk of bias [33]. Nevertheless, our overall risk of bias assessments was in agreement. Finally, the iCAT_SR tool and its guidance were used for a systematic assessment of intervention complexity [22], which facilitated an in‐depth understanding of MI interventions and enhanced the reporting and interpretation of our review findings. However, we did not conduct the iCAT_SR assessment independently by two reviewers.

## CONCLUSIONS

Overall, the evidence on MI appears inconclusive for improvements in medication adherence to prevent recurrent stroke and TIA, and for better QoL and clinical outcomes. This might be due to the short follow‐up periods, the ceiling effects on medication adherence, and the complex and heterogeneous MI interventions. Therefore, studies with longer follow‐up periods, validated measures, adequate sample sizes, and study populations meeting the general level of medication adherence in PwS are needed. In addition, future studies should consider the recommendations of the MRC framework to enhance the understanding of MI interventions, identify their successful elements, and refine them to achieve their intended effects.

## AUTHOR CONTRIBUTIONS

**Kathrin Wandscher:** Conceptualization; data curation; software; formal analysis; investigation; methodology; project administration; visualization; writing – original draft. **Falk Hoffmann:** Conceptualization; methodology; validation; writing – review and editing. **Christoph Heesen:** Writing – review and editing. **Götz Thomalla:** Writing – review and editing. **Anne Christin Rahn:** Conceptualization; methodology; writing – review and editing. **Jasmin Helbach:** Conceptualization; methodology; investigation; software; supervision; validation; writing – review and editing.

## FUNDING INFORMATION

This systematic review is part of the StrokeCompass project funded by the German Federal Ministry of Education and Research (grant number: 01GY2107). Open access funding was enabled and organized by Projekt DEAL.

## CONFLICT OF INTEREST STATEMENT

The authors declare that there is no conflict of interest.

## Supporting information


Appendix S1.

## Data Availability

The data that support the findings of this study are available in the supplementary material of this article.
