# Supplementary material for: Effectiveness of motivational interviewing on medication adherence for the prevention of recurrent stroke or transient ischemic attack: Systematic review of randomized controlled trials
Source: Eur J Neurol. 2024 Apr 27;31(8):e16313. doi: 10.1111/ene.16313 (PMC11235589; doi:10.1111/ene.16313)
Supplement: Supplementary file 1 — Appendix S1. [file ENE-31-e16313-s001.docx]

**Supplemental Material**

| Table S1. Search strategy……………………………………………………………………………………………………… | 2 |
| --- | --- |
| Table S2. Excluded records after full-text screening with reasons………………………………………… | 4 |
| Table S3. Detailed description of the intervention and comparator of the included studies… | 6 |
| Figure S1. Assessment of iCAT_SR dimensions of included studies……………………………………… | 8 |
| Table S4. Judgement of intervention complexity of the included studies with reasons using the iCAT_SR………………………………………………………………………………………………………………………. | 9 |
| Figure S2. Results of the risk of bias assessment of included studies using RoB 2………………… | 11 |
| Table S5. Judgement of risk of bias of included studies with reasons using the RoB 2 tool…… | 12 |
| Table S6. Outcomes and results for Quality of Life and clinical endpoints of the included studies………………………………………………………………………………………………………………………............ | 15 |
| References (supplemental material)…………………………………………………………………………………….. | 17 |

Table S1. Search strategy

| **Sources for search** | **Search terms** | |
| --- | --- | --- |
| ***Database (platform)*** | | |
| MEDLINE (PubMed)^†,‡^ | ("ischemic attack, transient"[mh] OR "Stroke"[mh] OR "Intracranial Hemorrhages"[mh] OR "TIA"[tiab] OR "transient ischemic attack*"[tiab] OR "transient ischaemic attack*"[tiab] OR "stroke"[tiab] OR "strokes"[tiab] OR "cerebrovascular accident*"[tiab] OR "CVA"[tiab] OR "apoplex*"[tiab] OR (("brain*"[tiab] OR "cerebr*"[tiab] OR "cerebell*"[tiab] OR "intracr*"[tiab] OR "intracerebr*"[tiab]) AND ("ischemi*"[tiab] OR "ischaemi*"[tiab] OR "infarct*"[tiab] OR "thromb*"[tiab] OR "embol*"[tiab] OR "occlus*"[tiab] OR "hypox*"[tiab])) OR (("brain*"[tiab] OR "cerebr*"[tiab] OR "cerebell*"[tiab] OR "intracr*"[tiab] OR "intracerebr*"[tiab] OR "intraventricular"[tiab] OR "subarachnoid*"[tiab]) AND ("haemorrhage*"[tiab] OR "hemorrhage*"[tiab] OR "haematoma*"[tiab] OR "hematoma*"[tiab] OR "bleed*"[tiab])) OR "SAH"[tiab]) AND ("Motivational Interviewing"[mh] OR "Directive Counseling"[mh] OR "Counseling"[mh] OR (("motivat*"[tiab]) AND ("interview*"[tiab] OR "session*"[tiab] OR "counsel*"[tiab] OR "therap*"[tiab]))) AND ("Randomized Controlled Trial"[pt] OR "Controlled Clinical Trial" [pt] OR "drug therapy" [sh] OR "random*"[tiab] OR "trial"[tiab] OR "control group"[tiab:~5] OR "control groups"[tiab:~5] OR "cluster RCT"[tiab:~3]) | |
| CINAHL (EBSCOhost)^†,‡^ | (MH "Cerebral Ischemia, Transient" OR MH "Stroke+" OR MH "Intracranial Hemorrhage+" OR TI ("TIA" OR "transient ischemic attack*" OR "transient ischaemic attack*") OR AB ("TIA" OR "transient ischemic attack*" OR "transient ischaemic attack*") OR TI ("stroke" OR "strokes" OR "cerebrovascular accident*" OR "CVA" OR "apoplex*") OR AB ("stroke" OR "strokes" OR "cerebrovascular accident*" OR "CVA" OR "apoplex*") OR ((TI "brain*" OR AB "brain*" OR TI "cerebr*" OR AB "cerebr*" OR TI "cerebell*" OR AB "cerebell*" OR TI "intracr*" OR AB "intracr*" OR TI "intracerebr*" OR AB "intracerebr*") AND (TI "ischemi*" OR AB "ischemi*" OR TI "ischaemi*" OR AB "ischaemi*" OR TI "infarct*" OR AB "infarct*" OR TI "thromb*" OR AB "thromb*" OR TI "embol*" OR AB "embol*" OR TI "occlus*" OR AB "occlus*" OR TI "hypox*" OR AB "hypox*")) OR ((TI "brain*" OR AB "brain*" OR TI "cerebr*" OR AB "cerebr*" OR TI "cerebell*" OR AB "cerebell*" OR TI "intracr*" OR AB "intracr*" OR TI "intracerebr*" OR AB "intracerebr*" OR TI "intraventricular" OR AB "intraventricular" OR TI "subarachnoid*" OR AB "subarachnoid*") AND (TI "haemorrhage*" OR AB "haemorrhage*" OR TI "hemorrhage*" OR AB "hemorrhage*" OR TI "haematoma*" OR AB "haematoma*" OR TI "hematoma*" OR AB "hematoma*" OR TI "bleed*" OR AB "bleed*")) OR TI "SAH" OR AB "SAH" ) AND (MH "Motivational Interviewing" OR MH "Counseling" OR ((TI "motivat*" OR AB "motivat*") AND (TI "interview*" OR AB "interview*" OR TI "session*" OR AB "session*" OR TI "counsel*" OR AB "counsel*" OR TI "therap*" OR AB "therap*"))) AND ( MH "Randomized Controlled Trials+" OR MH "Clinical Trials+" OR MH "Drug Therapy" OR TI ("random*" OR "trial") OR AB ("random*" OR "trial") OR AB (control W5 group*) OR (AB cluster W3 RCT)) | |
| PsycINFO (EBSCOhost)^†,§^ | (DE "Cerebral Ischemia" OR DE "Cerebral Infarction" OR DE "Cerebral Hemorrhage" OR DE "Subarachnoid Hemorrhage" OR TI ("TIA" OR "transient ischemic attack*" OR "transient ischaemic attack*") OR AB ("TIA" OR "transient ischemic attack*" OR "transient ischaemic attack*") OR TI ("stroke" OR "strokes" OR "cerebrovascular accident*" OR "CVA" OR "apoplex*") OR AB ("stroke" OR "strokes" OR "cerebrovascular accident*" OR "CVA" OR "apoplex*") OR ((TI "brain*" OR AB "brain*" OR TI "cerebr*" OR AB "cerebr*" OR TI "cerebell*" OR AB "cerebell*" OR TI "intracr*" OR AB "intracr*" OR TI "intracerebr*" OR AB "intracerebr*") AND (TI "ischemi*" OR AB "ischemi*" OR TI "ischaemi*" OR AB "ischaemi*" OR TI "infarct*" OR AB "infarct*" OR TI "thromb*" OR AB "thromb*" OR TI "embol*" OR AB "embol*" OR TI "occlus*" OR AB "occlus*" OR TI "hypox*" OR AB "hypox*")) OR ((TI "brain*" OR AB "brain*" OR TI "cerebr*" OR AB "cerebr*" OR TI "cerebell*" OR AB "cerebell*" OR TI "intracr*" OR AB "intracr*" OR TI "intracerebr*" OR AB "intracerebr*" OR TI "intraventricular" OR AB "intraventricular" OR TI "subarachnoid*" OR AB "subarachnoid*") AND (TI "haemorrhage*" OR AB "haemorrhage*" OR TI "hemorrhage*" OR AB "hemorrhage*" OR TI "haematoma*" OR AB "haematoma*" OR TI "hematoma*" OR AB "hematoma*" OR TI "bleed*" OR AB "bleed*")) OR TI "SAH" OR AB "SAH") AND (DE "Motivational Interviewing" OR DE "Counseling" OR ((TI "motivat*" OR AB "motivat*") AND (TI "interview*" OR AB "interview*" OR TI "session*" OR AB "session*" OR TI "counsel*" OR AB "counsel*" OR TI "therap*" OR AB "therap*"))) AND (DE "Clinical Trials" OR DE "Randomized Controlled Trials" OR DE "Drug Therapy" OR TI ("random*" OR "trial") OR AB ("random*" OR "trial") OR AB (control W5 group*) OR AB (cluster W3 RCT)) | |
| CENTRAL (Cochrane Library)^†^ | #1 | MeSH descriptor: [Ischemic Attack, Transient] this term only |
|  | #2 | MeSH descriptor: [Stroke] explode all trees |
|  | #3 | MeSH descriptor: [Intracranial Hemorrhages] explode all trees |
|  | #4 | "TIA":ti,ab OR "transient ischemic attack*":ti,ab OR "transient ischaemic attack*":ti,ab  OR "stroke":ti,ab OR "strokes":ti,ab OR "cerebrovascular accident*":ti,ab OR "CVA":ti,ab OR "apoplex*":ti,ab OR (("brain*":ti,ab OR "cerebr*":ti,ab OR "cerebell*":ti,ab OR "intracr*":ti,ab OR "intracerebr*":ti,ab) AND ("ischemi*":ti,ab OR "ischaemi*":ti,ab OR "infarct*":ti,ab OR "thromb*":ti,ab OR "embol*":ti,ab OR "occlus*":ti,ab OR "hypox*":ti,ab)) OR (("brain*":ti,ab OR "cerebr*":ti,ab OR "cerebell*":ti,ab OR "intracr*":ti,ab OR "intracerebr*":ti,ab OR "intraventricular":ti,ab OR "subarachnoid*":ti,ab) AND ("haemorrhage*":ti,ab OR "hemorrhage*":ti,ab OR "haematoma*":ti,ab OR "hematoma*":ti,ab OR "bleed*":ti,ab)) OR "SAH":ti,ab |
|  | #5 | #1 OR #2 OR #3 OR #4 |
|  | #6 | MeSH descriptor: [Motivational Interviewing] this term only |
|  | #7 | MeSH descriptor: [Directive Counseling] explode all trees |
|  | #8 | MeSH descriptor: [Counseling] explode all trees |
|  | #9 | "motivat*":ti,ab AND ("interview*":ti,ab OR "session*":ti,ab OR "counsel*":ti,ab OR "therap*":ti,ab) |
|  | #10 | #6 OR #7 OR #8 OR #9 |
|  | #11 | #5 AND #10 |
| ***Trial Register*** | | |
| ClinicalTrials.gov^¶^ |  | Condition or disease: “stroke” |
|  |  | Other terms: “Motivational interviewing” OR “counseling” |
|  |  | "Motivational interviewing" OR "counseling" \| "stroke" |
| Notes:  ^†^Search strategies used in other systematic reviews for stroke/TIA (Yang et al., 2013) and motivational interviewing (Zomahoun et al., 2017) were adapted.  ^‡^Search filters for identifying randomized trials developed by the Cochrane Collaboration for MEDLINE and CINAHL databases (Lefebvre et al., 2019) were added and adapted to each other.  ^§^Modified search filter for MEDLINE and CINAHL databases to identify randomized trials was added and adapted.  ^¶^Keywords from a previous review (Cheng et al., 2015) was reused. | | |

Table S2. Excluded records after full-text screening with reasons

| **Record** | **Reason for exclusion** |
| --- | --- |
| ***Database and register search*** | |
| Brouwer-Goossensen D, Genugten L, Lingsma H, Dippel D, Koudstaal P, Hertog H. The effectiveness of motivational interviewing on lifestyle behavior change after tia or minor ischemic stroke: a randomized controlled open label phase II trial. European Stroke Journal 2017;2:163. | 2 |
| Brouwer-Goossensen D, Scheele M, Genugten L van, Lingsma HF, Dippel DWJ, Koudstaal PJ, et al. Motivational interviewing in a nurse-led outpatient clinic to support lifestyle behaviour change after admission to a stroke unit: a randomized controlled trial. European Journal of Cardiovascular Nursing 2022;21:36–45. | 6 |
| Charite University of Berlin , Technical University of Munich, University of Erlangen-Nürnberg Medical School, München Praxis für Neurologie und Psychiatrie am Prinzregentenplatz, Technische Universität Berlin, Aarhus University Hospital, et al. Intensified Secondary Prevention Intending a Reduction of Recurrent Events in TIA and Minor Stroke Patients. Identifier: NCT01586702 [Internet]. 2011[cited 2023 Jun 12]. Available from: https://clinicaltrials.gov/study/NCT01586702 | 1 |
| Damush TM, Myers L, Anderson JA, Yu Z, Ofner S, Nicholas G, et al. The effect of a locally adapted, secondary stroke risk factor self-management program on medication adherence among veterans with stroke/TIA. Behav Med Pract Policy Res 2016;6:457–68. | 5 |
| Deans CF, Jack CIA, van den Broek M, Auton MF, Dickinson H, Forshaw D, et al. Evaluation of motivational interviewing early after acute stroke: a randomized controlled trial. Clinical Rehabilitation 2006;20:734–5. | 2 |
| Eichner FA, Schwarzbach CJ, Keller M, Haeusler KG, Hamann GF, er D, et al. Trial design and pilot phase results of a cluster-randomised intervention trial to improve stroke care after hospital discharge - The structured ambulatory post-stroke care program (SANO). Eur Stroke J 2021;6:213–21. | 3 |
| Flemming KD, Allison TG, Covalt JL, Herzig DE, Brown RD. Utility of a post-hospitalization stroke prevention program managed by nurses. Hosp Pract (1995) 2013;41:70–9. | 3 |
| Gianos E, Schoenthaler A, Mushailov M, Fisher EA, Berger JS. Rationale and design of the Investigation of Motivational Interviewing and Prevention Consults to Achieve Cardiovascular Targets (IMPACT) trial. Am Heart J 2015;170:430-7.e9. | 4 |
| Gillham S, Endacott R. Impact of enhanced secondary prevention on health behaviour in patients following minor stroke and transient ischaemic attack: a randomized controlled trial. Clin Rehabil 2010;24:822–30. | 6 |
| Green T, Haley E, Eliasziw M, Hoyte K. Education in stroke prevention: efficacy of an educational counselling intervention to increase knowledge in stroke survivors. Can J Neurosci Nurs 2007;29:13–20. | 6 |
| Hedegaard U, Kjeldsen LJ, Pottega˚rd A, Hallas J. A multifaceted pharmacist intervention to support medication adherence after stroke and transient ischemic attack. Int J Clin Pharm 2015; 37: 187. | 2 |
| Krishnamurthi R, Witt E, Barker-Collo S, McPherson K, Davis-Martin K, Bennett D, et al. Reducing recurrent stroke: methodology of the motivational interviewing in stroke (MIST) randomized clinical trial. International Journal of Stroke 2014;9:133‐139. | 1 |
| Laumeier I, Audebert HJ. Konzept der unterstützten Sekundärprävention nach Schlaganfall: Intensivierte Sekundärprävention mit der Intention einer Reduktion von Rezidivereignissen nach TIA- und Minor Stroke (INSPiRE-TMS) = Concept of supported secondary prevention after stroke: Intensified secondary prevention intending a reduction of recurrent events in TIA and minor stroke patients (INSPiRE-TMS). Nervenheilkunde: Zeitschrift Für Interdisziplinaere Fortbildung 2016;35:35–40. | 1 |
| Leiva A, Aguiló A, Fajó-Pascual M, Moreno L, Martín MC, Garcia EM, et al. Efficacy of a brief multifactorial adherence-based intervention in reducing blood pressure: a randomized clinical trial. Patient Prefer Adherence 2014;8:1683–90. | 4 |
| McManus JA, Craig A, McAlpine C, Langhorne P, Ellis G. Does behaviour modification affect post-stroke risk factor control? Three-year follow-up of a randomized controlled trial. Clinical Rehabilitation 2009;23:99–105. | 5 |
| McNaughton H, Weatherall M, McPherson K, Fu V, Taylor WJ, McRae A, et al. The effect of the Take Charge intervention on mood, motivation, activation and risk factor management: Analysis of secondary data from the Taking Charge after Stroke (TaCAS) trial. Clin Rehabil 2021;35:1021–31. | 5 |
| Odense University Hospital. Tailored Intervention to Improve Patient Adherence to Secondary Stroke Prevention Medication. Identifier: NCT01684176 [Internet]. 2012 [cited 2023 Jun 12]. Available from: https://clinicaltrials.gov/study/NCT01684176 | 1 |
| Watkins CL, Auton MF, Deans CF, Dickinson HA, Jack CI, Lightbody CE, et al. Motivational interviewing early after acute stroke: a randomized, controlled trial. Stroke 2007;38:1004–9. | 6 |
| Watkins CL, Wathan JV, Leathley MJ, Auton MF, Deans CF, Dickinson HA, et al. The 12-month effects of early motivational interviewing after acute stroke: a randomized controlled trial. Stroke 2011;42:1956–61. | 6 |
| ***Forward and backward citation searching*** | |
| Forster A, Young J. Specialist nurse support for patients with stroke in the community: a randomised controlled trial. BMJ 1996;312:1642–6. | 5 |
| Irewall A-L, Ögren J, Bergström L, Laurell K, Söderström L, Mooe T. Nurse-Led, Telephone-Based, Secondary Preventive Follow-Up after Stroke or Transient Ischemic Attack Improves Blood Pressure and LDL Cholesterol: Results from the First 12 Months of the Randomized, Controlled NAILED Stroke Risk Factor Trial. PLoS ONE 2015;10:e0139997. | 5 |
| Irewall A-L, Ulvenstam A, Graipe A, Ögren J, Mooe T. Nurse-based secondary preventive follow-up by telephone reduced recurrence of cardiovascular events: a randomised controlled trial. Sci Rep 2021;11:15628. | 5 |
| Joubert J, Davis SM, Donnan GA, Levi C, Gonzales G, Joubert L, et al. ICARUSS: An effective model for risk factor management in stroke survivors. International Journal of Stroke 2020;15:438–53. | 5 |
| Kono Y, Yamada S, Yamaguchi J, Hagiwara Y, Iritani N, Ishida S, et al. Secondary Prevention of New Vascular Events with Lifestyle Intervention in Patients with Noncardioembolic Mild Ischemic Stroke: A Single-Center Randomized Controlled Trial. Cerebrovasc Dis 2013;36:88–97. | 5 |
| Machline-Carrion MJ, Soares RM, Damiani LP, Campos VB, Sampaio B, Fonseca FH, et al. Effect of a Multifaceted Quality Improvement Intervention on the Prescription of Evidence-Based Treatment in Patients at High Cardiovascular Risk in Brazil: The BRIDGE Cardiovascular Prevention Cluster Randomized Clinical Trial. JAMA Cardiol 2019;4:408. | 5 |
| McAlister FA, Majumdar SR, Padwal RS, Fradette M, Thompson A, Buck B, et al. Case management for blood pressure and lipid level control after minor stroke: PREVENTION randomized controlled trial. CMAJ 2014;186:577–84. | 5 |
| Olaiya MT, Cadilhac DA, Kim J, Nelson MR, Srikanth VK, Gerraty RP, et al. Community-Based Intervention to Improve Cardiometabolic Targets in Patients with Stroke: A Randomized Controlled Trial. Stroke 2017;48:2504–10. | 5 |
| Rochette A, Korner-Bitensky N, Bishop D, Teasell R, White CL, Bravo G, et al. The YOU CALL–WE CALL Randomized Clinical Trial: Impact of a Multimodal Support Intervention After a Mild Stroke. Circ: Cardiovascular Quality and Outcomes 2013;6:674–9. | 5 |
| Teuschl Y, Matz K, Firlinger B, Dachenhausen A, Tuomilehto J, Brainin M, et al. Preventive effects of multiple domain interventions on lifestyle and risk factor changes in stroke survivors: Evidence from a two-year randomized trial. International Journal of Stroke 2017;12:976–84. | 5 |
| Willeit P, Toell T, Boehme C, Krebs S, Mayer-Suess L, Lang C, et al. STROKE-CARD care to prevent cardiovascular events and improve quality of life after acute ischaemic stroke or TIA: A randomised clinical trial. EClinicalMedicine 2020;25:100476. | 5 |
| Wolfe CDA, Redfern J, Rudd AG, Grieve AP, Heuschmann PU, McKevitt C. Cluster Randomized Controlled Trial of a Patient and General Practitioner Intervention to Improve the Management of Multiple Risk Factors After Stroke: Stop Stroke. Stroke 2010;41:2470–6. | 5 |
| Note: Reasons for exclusion:  1) No randomized controlled trial  2) Conference abstract  3) Sample size <50 participants  4) No participants with history of stroke or transient ischemic attack  5) No Motivational Interviewing  6) Medication adherence not reported  The assignment of the reasons for exclusion was done hierarchically in the order presented. |  |

Table S3. Detailed description of the intervention and comparator of the included studies

| **Authors (year)** | **Components of intervention** | **Content of MI** | **Intervention provider (qualification, expertise) / Training (standardized approach)** | **Number of sessions, type of delivery (session duration, adaptions), intervention duration (time points)** | **Control group** |
| --- | --- | --- | --- | --- | --- |
| Ahmadi et al. (2020) with companion report: Ihl et al. (2022)^†^ | The intervention included: (1) assessment of risk factors, (2) counseling with feedback and MI strategies, (3) summarized recommendations in a written report in a language comprehensible to the patient, (4) information about complementary resources (e.g., physical activity or smoking cessation programs), (5) education about stroke risk factors, risk of recurrence, and risk reduction, (6) contact with German GPs in complex cases, for critical findings or interdisciplinary consensus. | The counseling was based on the risk factor assessment using MI to collaboratively elaborate a personalized plan for targeted risk factor modification according to current guideline recommendations addressing both medication adherence and lifestyle changes^‡^. | The intervention was prepared by trained nurses or health-care assistants (not reported) through risk factor assessment and provided by physicians (board-certified, expertise in neurovascular medicine). / Standardized MI training over 2 days with scheduled 1 day refresh course after 6 months for physicians^‡^ and nurses^§^ (not reported). | A total of 8 MI sessions, all conducted as face-to-face interviews (session duration: not reported, involving relatives if possible) in outpatient clinics, over 24 months (3, 6, and 12 weeks and 6, 9, 12, 18, and 24 months after randomization). | Usual Care |
| Barker-Collo et al. (2015) | The intervention included: (1) audio-recorded MI sessions incorporating consideration of readiness for change based on TTM and commitment to change by defining SMART goals^¶^. | The MI sessions were used to understand the status quo of the patient's behavior, values, beliefs, and visions, which were used in subsequent sessions to motivate for change and collaboratively set goals for future risk factor modification, addressing both medication adherence and lifestyle changes^¶^. Information on CVD risk and prevention were provided only on patients request in these sessions^#^. | The intervention was provided under the supervision of MI trainers by trained researchers (not reported), who remained consistent when possible. / Education in stroke risk factors and training in MI according to the key principles of Miller and Rollnick by an experienced trainer who provided ongoing individual or group training and feedback throughout the study (an intervention guideline for each time point was developed, as well as tools to assist the interview process)^#^. | A total of 4 MI sessions, an initial face-to-face interview at the hospital or patient's primary location (60-90 minutes, adaptions: not reported) and 3 subsequent telephone interviews (à 30 minutes, can also be conducted as face-to-face sessions at the patient's primary location upon request or in the case of hearing impairments), over 9 months (4 weeks, 3, 6, and 9 months after stroke). | Usual Care |
| Hedegaard et al. (2014) | The intervention included: (1) medication review of preventative medication, (2) audio-recorded patient interviews based on MI including educational material^¶^, (3) follow-up telephone interviews addressing medication problems, (4) written summary of each session, including the patient's stated goals and a collaborative plan. | The patient interview was based on the medication review using MI to address the status quo of medication intake, beliefs, and issues raised by the patient with a tailored toolbox including MI tools, adherence aids, and VisualRx plots with a primary focus on medication adherence^¶^. In the telephone interviews, MI was performed if necessary^¶^. | The intervention was delivered by ~~clinical~~ pharmacists (master's degree, 1.5 to 15 years of hospital practice experience)^¶^. / Education in stroke pathology and treatment, and MI training consisting of a 3-day course on the key principles of Miller and Rollnick, 1 week of interview training, and an 1-day MI follow-up course one month after the beginning of the study (an interview guideline and an intervention guide were developed)^¶^. | A total of 4 sessions, an initial face-to-face MI interview in the hospital (20-30 minutes, allowing relatives to participate)^¶^ and 3 subsequent telephone interviews (à 15 minutes, adaptions: not reported), over 6 months (at least 3 days before discharge, 1 week, 2 and 6 months after discharge). | Usual care |
| Mackenzie et al. (2013) | The intervention of a nurse-led case management program included: (1) telephone interviews based on MI (2) home BP monitoring, (3) use of dosettes for medication administration. | During the telephone interviews, MI was used to promote risk factor reduction addressing medication adherence and BP management. | The intervention was delivered by NPs and CNSs (qualification: not reported, prior education in MI according to Canadian stroke prevention strategy). / Not reported (a sample of MI scripts were developed by 1 CNS). | Not reported, MI telephone interviews (not reported), over 6 months (at least 1 interview per month after randomization). | Usual Care |
| Abbreviations: **BP**: Blood pressure; **CNS**: Clinical nurse specialists; **CVD**: Cardiovascular disease; **GP**: General practitioner; **MI**: Motivational Interviewing; **NP**: Nurse practitioner; **SMART**: Specific, Measurable, Achievable, Relevant, and Time-Bound; **SPC**: Stroke prevention clinic; **TTM**: Transtheoretical model.  Notes:  ^†^Data were obtained from the report of Ahmadi et al. (2020) unless otherwise indicated.  ^‡^Data obtained from the referred study protocol by Leistner et al. (2013).  ^§^MI training for nurses was reported only by Ahmadi et al. (2020) and not by Ihl et al. (2022) or the study protocol by Leistner et al. (2013).  ^¶^Additional data from supplementary material.  ^#^Data obtained from the referred study protocol by Krishnamurthi et al. (2014). | | | | | |


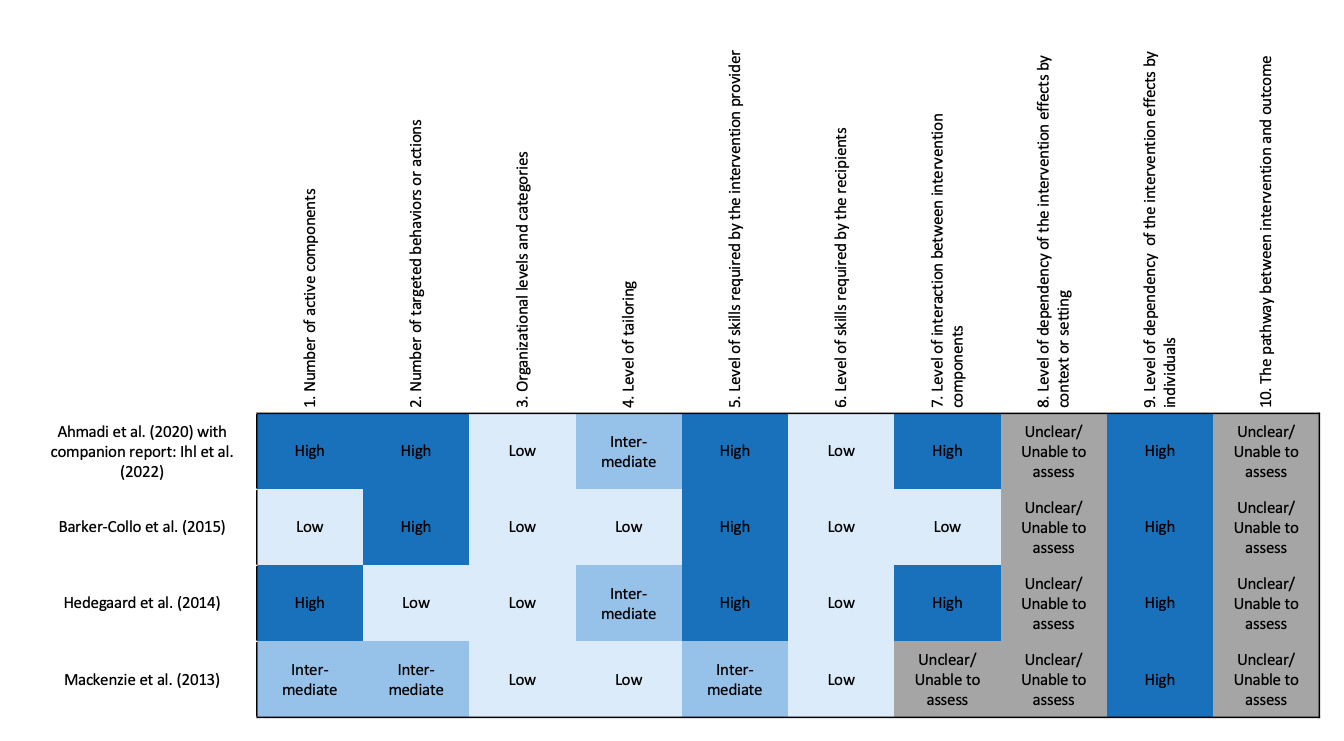


Figure S2. Assessment of iCAT_SR dimensions of included studies. The intervention complexity is graded as "high", "intermediate", "low", "varies" or "unclear/unable to assess" according to categorization of Cadogan et al. (2020).

Table S4. Judgement of intervention complexity of the included studies with reasons using the iCAT_SR

| **iCAT_SR dimensions** | **Authors (year)** | | | |
| --- | --- | --- | --- | --- |
|  | Ahmadi et al. (2020) with companion report: Ihl et al. (2022)^†^ | Barker-Collo et al. (2015) | Hedegaard et al. (2014) | Mackenzie et al. (2013) |
| **Core dimensions** | | | | |
| 1. Active components included in the intervention, in relation to the comparison | *More than one component and delivered as a bundle:* More than five components that had to be delivered in a subsequent order (p. 3-4). | *One component:* MI is the only active component that integrates readiness for change based on TTM and SMART goals (supplemental material p. 13). | *More than one component and delivered as a bundle:* More than four components, with medication review required before initial interview (p. 223). | *More than one component:* A total of four components, the necessity of a particular order is not described (p. 29). |
| 2. Behavior or actions of intervention recipients or participants to which the intervention is directed | *Multi-target:* Intervention targets different behaviors (e.g., smoking cessation, regular physical activity, healthy diet, and medication adherence) (p. 3). | *Multi-target:* Intervention targets medication adherence, nutrition, and physical activity ( Krishnamurthi et al., [2014], p. 136)^‡^. | *Single target:* Intervention primarily targets medication adherence, lifestyle may be an issue raised by participants (supplemental material p. 3). | *Dual target:* Intervention targets participants' medication adherence and blood pressure goals (p. 28). |
| 3. Organizational levels and categories targeted by the intervention | *Single category:* Intervention is mediated through HCPs, but it is directed to participants (p. 3-4). | *Single category:* Intervention is mediated through researchers, but it is directed to participants (p. 3454). | *Single category:* Intervention is mediated through clinical pharmacists, but it is directed to participants (p. 223). | *Single category:* Intervention is mediated through nurse case managers, but it is directed to participants (p. 28). |
| 4. The degree of tailoring intended, or flexibility permitted across sites or individuals in applying or implementing the intervention | *Moderately tailored: P*hysician-led counseling, as well as contact with GPs, are tailored to the patient's risk factors and relatives were involved, when possible, but the components need to be delivered in the intervention facilities and in a subsequent order (p. 3-4). | *Inflexible:* Although the delivery of MI could be tailored (face-to-face or telephone), the intervention manual is highly standardized, allowing only limited variation (Krishnamurthi et al., [2014], p. 136^‡^; supplemental material p. 3-15). | *Moderately tailored:* In the first interview, the toolbox was tailored to the specific needs of the participants and relatives were allowed to participate, with MI performed in later interviews as needed, but the intervention had to follow the standardized guideline (supplemental material p. 2-4). | *Inflexible:* The monthly call may be more frequent if needed, but no further tailoring or flexibility is reported (p. 29). |
| 5. The level of skill required by those delivering the intervention in order to meet the intervention objectives | *High level skills:* While the feedback strategies are small extensions of the HCP's existing skills, the new skill of MI is an addition and required extensive additional training (p. 3-4). | *High level skills:* Extensive additional training was required to train researchers in MI and stroke risk factors (Krishnamurthi et al., [2014], p. 136)^‡^. | *High level skills:* Extensive additional training was required to train pharmacists in MI and stroke pathology and treatment, while the medication review is a small extension of their existing skills (supplemental material p. 1). | *Intermediate level skills:*  The nurse case managers had prior skills in MI, and the MI scripts developed are considered a small extension of existing skills in facilitating intervention delivery (p. 28). |
| 6. The level of skill required for the targeted behavior when entering the included studies by those receiving the intervention (consumers, professionals, planners) in order to meet the intervention objectives | *Basic skills:* Participation in the study did not require any special skills (p. 2-3). | *Basic skills:* Participation in the study did not require any special skills (p. 3453). | *Basic skills:* Participation in the study did not require any special skills (p. 223). | *Basic skills:* Participation in the study did not require any special skills (p. 28). |
| **Optional dimensions** | | | | |
| 7. The degree of interaction between intervention components, including the independence/interdependence of intervention components | *High level interaction:* The interdependency is temporal between risk factor assessment and the physician-led counseling, resulting in a synergistic effect (p. 3-4). | *Independent:* The intervention only consists of MI as one component (p. 3454). | *High level interaction:* The interdependency is temporal between the medication review and the first interview, resulting in a synergistic effect (p. 223). | *Unclear/unable to assess:* The intervention consists of different components, but the independence or interdependence is not described (p. 29). |
| 8. The degree to which the effects of the intervention are dependent on the context or setting in which it is implemented | *Unclear/unable to assess:* The intervention was provided in hospitals and in a stroke center in two countries, but the effects on the outcome are not shown separately (p. 6-10). | *Unclear/unable to assess:* The intervention was delivered only in the hospital setting in a specific region, which did not allow for consideration of potential interactions between the intervention and different settings  (Krishnamurthi et al., [2014], p. 134)^‡^. | *Unclear/unable to assess:* The intervention was delivered in only one hospital in a specific region, which includes both inpatient and outpatient care, but the effects on the outcome are not shown separately for the different settings (p. 223). | *Unclear/unable to assess:* The intervention was delivered only in nurse-led stroke prevention clinics in a specific region, which did not allow for consideration of potential interactions between the intervention and different settings (p. 28). |
| 9. The degree to which the effects of the intervention are changed by recipient or provider factors | *Highly dependent on individual-level factors:* The intervention incorporates MI as a form of counseling that is therapist-dependent, resulting in both the provider and the recipient modifying the effects of the intervention (p. 3-4). | *Highly dependent on individual-level factors:* The intervention incorporates MI as a form of counseling that is therapist-dependent, resulting in both the provider and the recipient modifying the effects of the intervention (p. 3454). | *Highly dependent on individual-level factors:* The intervention incorporates MI as a form of counseling that is therapist-dependent, resulting in both the provider and the recipient modifying the effects of the intervention (p. 223). | *Highly dependent on individual-level factors:* The intervention incorporates MI as a form of counseling that is therapist-dependent, resulting in both the provider and the recipient modifying the effects of the intervention (p. 28). |
| 10. The nature of the causal pathway between the intervention and the outcome it is intended to effect | *Unclear/unable to assess:* The pathway by which the intervention effects the outcome is neither narratively described nor graphically visualized (Leistner et al., [2013] p. 2-5^‡^; p. 3-4). | *Unclear/unable to assess:* The pathway by which the intervention effects the outcome is neither narratively described nor graphically visualized (Krishnamurthi et al., [2014], p. 134-137^‡^; p. 3452- 3455). | *Unclear/unable to assess:* The pathway by which the intervention effects the outcome is neither narratively described nor graphically visualized (p. 222-225; supplemental material p. 1-4). | *Unclear/unable to assess:* The pathway by which the intervention effects the outcome is neither narratively described nor graphically visualized (p. 28-29). |
| Abbreviations: **CVD**: Cardiovascular disease **GP**: General practitioner; **HCP**: Healthcare professional; **iCAT_SR**: Intervention Complexity Assessment Tool for Systematic Reviews **; MI:** Motivational Interviewing; **SMART**: Specific, Measurable, Achievable, Relevant, and Time-Bound; **TTM**: Transtheoretical model.  Notes:  ^†^Data were obtained from the report of Ahmadi et al. (2020) unless otherwise indicated.  ^‡^Data obtained from referred study protocol. | | | | |


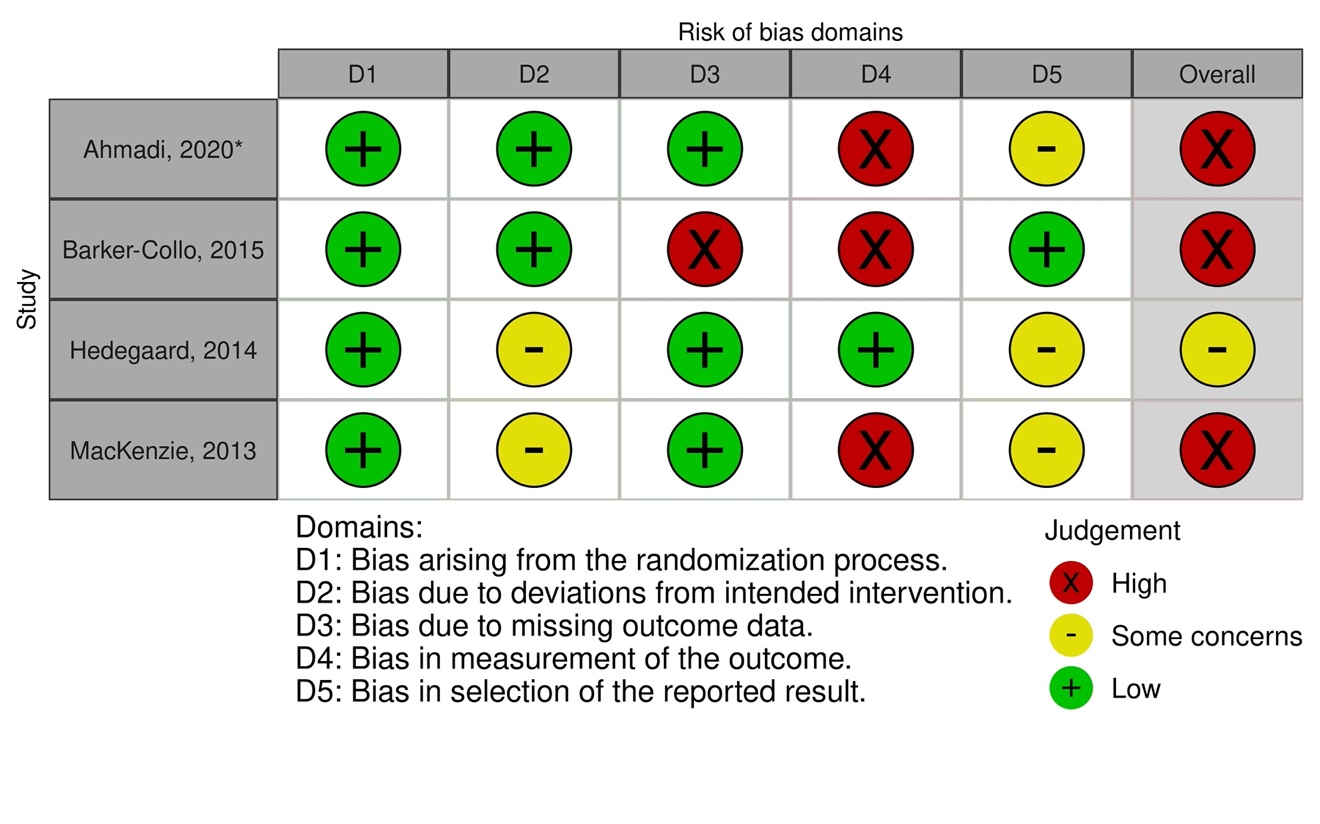


Figure S2. Results of the risk of bias assessment of included studies using RoB 2 (Sterne et al., 2019). *The article by Ihl et al. (2022) was excluded for risk of bias assessment as medication adherence was not measured.

Table S5. Judgement of risk of bias of included studies with reasons using the RoB 2 tool

| **RoB 2 domains** | **Authors (year)** | | | |
| --- | --- | --- | --- | --- |
|  | Ahmadi et al. (2020)^†^ | Barker-Collo et al. (2015) | Hedegaard et al. (2014) | Mackenzie et al. (2013) |
| **Domain-level risk-of-bias judgements^‡^** | | | | |
| **Randomization process** | | | | |
| Judgement | Low | Low | Low | Low |
| Support for judgement | A random component was used in the allocation sequence, the process was concealed by an external unit, and there were no baseline imbalances. | A random component was used in the allocation sequence, the process was concealed by an remote method, and there were no baseline imbalances. | A random component was used in the allocation sequence, the process was concealed by appropriately used envelopes, and there were no baseline imbalances. | A random component was used in the allocation sequence, the process was concealed by an external unit, and there were no baseline imbalances. |
| Accompanied citation | “Patients were randomly assigned (1:1) with a secure web-based computer­generated block randomisation procedure (block size of 3), provided by the Charité Coordination Center for Clinical Studies […]”(p. 3). | “Eligible study participants are randomized to either the MI or UC groups using web-based computerized randomization software” (Krishnamurthi et al., [2014], p. 134)^§^. | “Randomization block sizes of 4 and 6 were generated by a computerized random number generator prior to enrollment, and the allocation was concealed in numbered opaque envelopes” (p. 225). | “A centralized telephone randomization system was initiated with […] random blocking tables to provide even distribution of intervention and usual care subject assignment at each of the four sites” (p. 29). |
| **Deviations from the intended interventions (effect of assignment to intervention)** | | | | |
| Judgement | Low | Low | Some concerns | Some concerns |
| Support for judgement | Participants and intervention providers were unblinded, without deviating from the intended intervention, and an ITT analysis was used. | Participants and intervention providers were unblinded, without deviating from the intended intervention, and an ITT analysis was used. | Participants and intervention providers were unblinded, without deviating from the intended intervention, and a PP analysis was used. | Participants and intervention providers seemed to be unblinded, with no information about deviation from the intended intervention and no information about the method of analysis used. |
| Accompanied citation | “Neither patients nor intervention staff were masked to treatment allocation” (p.3). | “[…] study participants are requested not to disclose their group allocation by mentioning any contact with the motivational interviewer to the RA [research assistant] conducting the assessment” (Krishnamurthi et al., [2014], p. 136)^§^. | “We conducted an exploratory per-protocol analysis” (p. 225). | “Participants in the intervention group received […] monthly telephone follow-up by advanced practice nurses using motivational interviewing techniques to promote risk factor reduction over a period of six months” (p. 29). |
| **Missing outcome data** | | | | |
| Judgement | Low | High | Low | Low |
| Support for judgement | Data were available for nearly all randomized participants. | Data were not available for more than 5% of all randomized participants, with different proportions of withdrawals between the intervention and control group, with no clear evidence that the results are not biased and no documented reasons for loss to follow-up or withdrawal that could have influenced the missingness of the outcome by its true value. | Data were available for nearly all randomized participants. | Data were available for all randomized participants. |
| Accompanied citation | "1030 (98·3%) of the support programme group and 1042 (99·2%) patients of the conventional care group were included in the intention­to­treat analysis […]” (p. 6). | In the flow chart, attrition was reported only as lost to follow-up or withdrawal without specified reason. In addition, the availability of self-reported adherence data ranged from 172 to 159 participants in the IG and 176 to 161 participants in the CG for a total of 193 randomized participants in each group (p. 3452-3454). | “Of the 211 randomized patients, 8 were excluded from the primary analyses […] as adherence could not be estimated according to the protocol” (p. 226-227). | “All 56 participants completed the study” (p. 29). |
| **Measurement of the outcome** | | | | |
| Judgement | High | High | Low | High |
| Support for judgement | There was no specific information on the outcome measure, the time points were comparable, a self-report measure was used with knowledge of the assigned intervention and could have influenced the outcome assessment. | The outcome measure seemed appropriate, the time points and measures were comparable, a self-report measure was used with knowledge of the assigned intervention and could have influenced the outcome assessment. | The outcome measures were appropriate, the time points and measures were comparable, objective measures were used, and the outcome assessors were blinded to the intervention. | The outcome measures seemed appropriate, the time points and measures were comparable, self-report measures were used, and participants were aware of the assigned intervention, which could have influenced the outcome assessment. |
| Accompanied citation | “Endpoints will be assessed in a written questionnaire that will be handed out to the patients prior to further follow-up assessments” (Leistner et al., [2013], p. 5)^§^. | “[…] self-reported adherence to prescribed medications, including self-reported use of antiplatelet/anticoagulant medications, statin, and blood pressure-lowering therapy medications as prescribed (and crosschecked with electronic medication dispense records, where available)” (Krishnamurthi et al., [2014], p. 136)^§^. | “The adherence assessment was based on the medication possession ratio (MPR) measure […]” (p. 224). | “Six months following recruitment, research assistants at each site visited the participants either at their home or at the clinic to measure post intervention […] self-reported adherence” (p. 29). |
| **Selection of reported results** | | | | |
| Judgement | Some concerns | Low | Some concerns | Some concerns |
| Support for judgement | The data were analyzed and measured according to a pre-specified plan and were comparable to those reported in the study protocol, with no information on the measurement of medication adherence as an outcome and without selection or multiple analyses of results. | The data were analyzed and measured according to a pre-specified plan and were comparable to those reported in the study protocol, with only one outcome measure and no selected results from multiple analyses of the data. | The data were not measured according to a pre-specified plan without justification and are therefore not comparable to those reported in the trial registration, with multiple outcome measures of medication adherence and no selected results from multiple analyses of the data. | There was neither a trial registration nor a study protocol for comparisons, and the statistical analysis was not reported in sufficient detail to identify a pre-specified plan. |
| Accompanied citation | “We did all statistical analyses in accordance with the prespecified statistical analysis plan” (p. 5). | Self-reported adherence was assessed as planned (p. 3453) “[…] at 28 days, three-, six-, nine- and 12 months following stroke […]” (Krishnamurthi et al., [2014], p. 136)^§^. | Medication adherence was planned to be measured by the proportion of days covered (Odense University Hospital, outcome measures section, [2012])^¶^ but was later measured by the medication possession ratio (p. 224). | No statistical methods were reported (p. 28-29). |
| **Overall risk-of-bias judgement^b^** | | | | |
| **Overall bias** | | | | |
| Judgement | High | High | Some concerns | High |
| Support for judgement | The study is judged to be at high risk of bias in one domain and some concerns in another domain for this result. | The study is judged to be at high risk of bias in two domains for this result. | The study is judged to raise some concerns in two domains for this result but is not judged to be at high risk of bias in any other domain. | The study is judged to be at high risk of bias in one domain and some concerns in two other domains for this result. |
| Abbreviations: **CG**: Control group; **N.R.**: Not reported; **PP**: Per-protocol; **IG**: Intervention group; **ITT**: Intention-to-treat; **RoB 2**: Second version of the Cochrane risk-of-bias tool for randomized trials.  Notes:  ^†^The article by Ihl et al. (2022) was not included in the risk of bias assessment because no medication adherence outcomes were measured.  ^‡^The results of the risk of bias assessments followed the provided algorithm or criteria of the RoB 2 tool and were not overridden by the assessors.  ^§^Data obtained from referred study protocol.  ^¶^Data obtained from referred trial registration. | | | | |

Table S6. Outcomes and results for Quality of Life and clinical endpoints of the included studies

| **Authors (year)** | **Secondary outcomes: QoL and clinical endpoints (measurement)** | **Effect on QoL and clinical endpoints** |  |
| --- | --- | --- | --- |
| Ahmadi et al. (2020) with companion report: Ihl et al. (2022)^†^ | **3-P-MACE** (discharge letters, death certificates, and clinical documentation), **all-cause deaths** (death certificates), **recurrence of stroke** (discharge letters and clinical documentation), **hospitalization due to major cardiovascular events or other cardiovascular events** (discharge letters and clinical documentation), **QoL** (EQ5D-3L). | **12 months:**  EQ5D-3L: IG (n=822): 0.824 ± 0.230 vs. CG (n=845): 0.811 ± 0.242, 0.013 MD (95% CI: -0.009 to 0.036)  **24 months:**  EQ5D-3L: IG (n= 687): 0.793 ± 0.273 vs. CG (n=685): 0.790 ± 0.270, 0.003 MD (95% CI: -0.026 to 0.032)  **36 months:**  EQ5D-3L: IG (n= 565): 0.773 ± 0.292 vs. CG (n=558): 0.770 ± 0.308, 0.003 MD (95% CI: -0.032 to 0.038)  **60 months:**  3-P-MACE: IG (n=1030): 15.8% vs. CG (n=1042): 16.8%, HR 0.92 (95% Cl: 0.75 to 1.14)  Recurrence of stroke: IG (n=1030): 11.8% vs. CG (n=1042): 11.4%, HR 1.02 (95% Cl: 0.79 to 1.32)  All-cause deaths: IG: (n=1030): 7.1% vs. CG (n=1042): 8.2%, HR 0.85 (95% Cl: 0.62 to 1.17)  Hospitalization for major vascular events: IG: 330/1000 PY vs. 359/1000 PY, IRR 0.92 (95% Cl: 0.79 to 1.07)  Hospitalization for other vascular events: IG: 158/1000 PY vs. 164/1000 PY, IRR 0.97 (95% Cl: 0.78 to 1.20) |  |
| Barker-Collo et al. (2015) | **Recurrence of stroke or TIA** (not reported), **QoL** (SF-36, producing a PCS and a MCS). | **3 months:**  SF-36:  *PCS:* IG (n=172): 40.64 ± 10.82 vs. CG: (n=179): 41.75 ± 11.03, -1.30 MD (95% CI: -3.46 to 0.85)  *MCS:* IG (n=172): 50.56 ± 10.67 vs. CG: (n=179): 50.79 ± 9.85, -0.44 MD (95% CI: -2.60 to 1.72)  **6 months:**  SF-36:  *PCS:* IG (n=176): 42.16 ± 12.00 vs. CG: (n=175): 41.97 ± 11.59, -0.16 MD (95% CI: -2.31 to 1.99)  *MCS:* IG (n=176): 50.32 ± 11.54 vs. CG: (n=175): 51.70 ± 10.04, -1.29 MD (95% CI: -3.44 to 0.87)  **9 months:**  SF-36:  *PCS:* IG (n=161): 41.70 ± 11.51 vs. CG: (n=165): 41.03 ± 11.71, 0.08 MD (95% CI: -2.10 to 2.26)  *MCS:* IG (n=161): 51.99 ± 10.39 vs. CG: (n=165): 51.69 ± 10.38, 0.22 MD (95% CI: -1.98 to 2.43)  **12 months:**  SF-36:  *PCS:* IG (n=165): 43.02 ± 10.88 vs. CG: (n=169): 43.52 ± 10.73, -0.59 MD (95% CI: -2.75 to 1.57)  *MCS:* IG (n=165), 51.93 ± 10.98 vs. CG: (n=169): 52.47 ± 8.84, -0.68 MD (95% CI: -2.86 to 1.49)  Recurrence of stroke: IG (n=193): 2.1% vs. CG (n=193): 3.1%, RR 0.667 (95% CI: 0.19 to 2.33)  Recurrence of TIA: IG (n=193): 2.1% vs. CG (n=193): 2.1%, RR 1.000 (95% CI: 0.25 to 3.94) |  |
| Hedegaard et al. (2014) | **3-P-MACE** (Danish National eHealth Portal, EMRs and Danish Civil Registration system). | **12 months:**  3-P-MACE: IG (n=102): 5.0% vs. CG (n=101): 5.0%, RR 0.99 (95% CI: 0.30 to 3.32) |  |
| Mackenzie et al. (2013) | **Recurrence of stroke or TIA** (SPC and hospital readmission documentation). | **6 months:**  Recurrence of stroke: IG (n=29): 6.9% vs. CG (n=27): 0%^‡^ (CI or p-value not reported) |  |
| Abbreviations: **±**: Standard deviation; **3-P-MACE**: Composite endpoint of stroke, myocardial infarction and cardiovascular deaths; **CG**: Control group; **CI**: Confidence Interval; **EMR**: Electronic medical records**; EQ-5D-3L**: European Quality of Life 5 Dimension 3 Level; **HR:** Hazard Ratio; **IG**: Intervention group; **IRR**: Incidence rate ratio; **MCS**: Mental component score; **QoL:** Quality of life; **PCS:** Physical component score; **PY**: Patient-years; **RR**: Risk ratio; **SF-36**: 36-item Short Form Health Survey; **SPC**: Stroke prevention clinic; **TIA**: Transient ischemic attack.  Notes:  ^†^QoL measure and its results were obtained from the companion report by Ihl et al. (2022).  ^‡^Percentage proportion was not directly reported and had to be self-calculated based on numbers provided by authors in the text (IG: 2/29 = 0.069; CG: 0/27= 0). | | | |

**References (supplemental material)**

Ahmadi, M., Laumeier, I., Ihl, T., Steinicke, M., Ferse, C., Endres, M., Grau, A., Hastrup, S., Poppert, H., Palm, F., Schoene, M., Seifert, C. L., Kandil, F. I., Weber, J. E., von Weitzel-Mudersbach, P., Wimmer, M. L. J., Algra, A., Amarenco, P., Greving, J. P., Busse, O., … Audebert, H. J. (2020). A support programme for secondary prevention in patients with transient ischaemic attack and minor stroke (INSPiRE-TMS): an open-label, randomised controlled trial. The Lancet. Neurology, 19(1), 49–60. https://doi.org/10.1016/S1474-4422(19)30369-2

Barker-Collo, S., Krishnamurthi, R., Witt, E., Feigin, V., Jones, A., McPherson, K., Starkey, N., Parag, V., Jiang, Y., Barber, P. A., Rush, E., Bennett, D., & Aroll, B. (2015). Improving Adherence to Secondary Stroke Prevention Strategies Through Motivational Interviewing: Randomized Controlled Trial. Stroke, 46(12), 3451–3458. https://doi.org/10.1161/STROKEAHA.115.011003

Cadogan, C. A., Rankin, A., Lewin, S., & Hughes, C. M. (2020). Application of the intervention Complexity Assessment Tool for Systematic Reviews within a Cochrane review: an illustrative case study. HRB open research, 3, 31. https://doi.org/10.12688/hrbopenres.13044.1

Cheng, D., Qu, Z., Huang, J., Xiao, Y., Luo, H., & Wang, J. (2015). Motivational interviewing for improving recovery after stroke. The Cochrane database of systematic reviews, 2015(6), CD011398. https://doi.org/10.1002/14651858.CD011398.pub2

Ihl, T., Ahmadi, M., Laumeier, I., Steinicke, M., Ferse, C., Klyscz, P., Endres, M., Hastrup, S., Poppert, H., Palm, F., Kandil, F. I., Weber, J. E., von Weitzel-Mudersbach, P., Wimmer, M. L. J., & Audebert, H. J. (2022). Patient-Centered Outcomes in a Randomized Trial Investigating a Multimodal Prevention Program After Transient Ischemic Attack or Minor Stroke: The INSPiRE-TMS Trial. Stroke, 53(9), 2730–2738. https://doi.org/10.1161/STROKEAHA.120.037503

Hedegaard, U., Kjeldsen, L. J., Pottegård, A., Bak, S., & Hallas, J. (2014). Multifaceted intervention including motivational interviewing to support medication adherence after stroke/transient ischemic attack: a randomized trial. *Cerebrovascular diseases extra*, *4*(3), 221–234. https://doi.org/10.1159/000369380

Krishnamurthi, R., Witt, E., Barker-Collo, S., McPherson, K., Davis-Martin, K., Bennett, D., Rush, E., Suh, F., Starkey, N., Parag, V., Rathnasabapathy, Y., Jones, A., Brown, P., Te Ao, B., Feigin, V. L., & ARCOS IV Programme Group (2014). Reducing recurrent stroke: methodology of the motivational interviewing in stroke (MIST) randomized clinical trial. International journal of stroke : official journal of the International Stroke Society, 9(1), 133–139. https://doi.org/10.1111/ijs.12107

Leistner, S., Michelson, G., Laumeier, I., Ahmadi, M., Smyth, M., Nieweler, G., Doehner, W., Sobesky, J., Fiebach, J. B., Marx, P., Busse, O., Köhler, F., Poppert, H., Wimmer, M. L., Knoll, T., Von Weitzel-Mudersbach, P., & Audebert, H. J. (2013). Intensified secondary prevention intending a reduction of recurrent events in TIA and minor stroke patients (INSPiRE-TMS): a protocol for a randomised controlled trial. BMC neurology, 13, 11. https://doi.org/10.1186/1471-2377-13-11

Lefebvre, C., Glanville, J., Briscoe, S., Featherstone, R., Littlewood, A., Marshall, C., Metzendorf, M. I., Noel-Storr, A., Paynter, R., Rader, T., Thomas, J., Wieland, L. S. (2022). Technical Supplement to Chapter 4: Searching for and selecting studies. In: Higgins, J. P. T., Thomas, J., Chandler, J., Cumpston, M., Li, T., Page, M. J., Welch, V. A. (editors). Cochrane Handbook for Systematic Reviews of Interventions version 6.3 (updated February 2022). Cochrane. Cited 2023 Apr 30, Available from www.training.cochrane.org/handbook

Mackenzie, G., Ireland, S., Moore, S., Heinz, I., Johnson, R., Oczkowski, W., & Sahlas, D. (2013). Tailored interventions to improve hypertension management after stroke or TIA--phase II (TIMS II). Canadian journal of neuroscience nursing, 35(1), 27–34.

Odense University Hospital (2012). Tailored Intervention to Improve Patient Adherence to Secondary Stroke Prevention Medication. Identifier: NCT01684176. Cited 2023 Jun 12, Available from: https://clinicaltrials.gov/study/NCT01684176

Sterne, J. A. C., Savović, J., Page, M. J., Elbers, R. G., Blencowe, N. S., Boutron, I., Cates, C. J., Cheng, H. Y., Corbett, M. S., Eldridge, S. M., Emberson, J. R., Hernán, M. A., Hopewell, S., Hróbjartsson, A., Junqueira, D. R., Jüni, P., Kirkham, J. J., Lasserson, T., Li, T., McAleenan, A., … Higgins, J. P. T. (2019). RoB 2: a revised tool for assessing risk of bias in randomised trials. BMJ (Clinical research ed.), 366, l4898. https://doi.org/10.1136/bmj.l4898

Yang, M., Cheng, H., Wang, X., Ouyang, M., Shajahan, S., Carcel, C., Anderson, C., Kristoffersen, E. S., Lin, Y., Sandset, E. C., Wang, X., & Yang, J. (2022). Antithrombotics prescription and adherence among stroke survivors: A systematic review and meta-analysis. Brain and behavior, 12(10), e2752. https://doi.org/10.1002/brb3.2752

Zomahoun, H. T. V., Guénette, L., Grégoire, J. P., Lauzier, S., Lawani, A. M., Ferdynus, C., Huiart, L., & Moisan, J. (2017). Effectiveness of motivational interviewing interventions on medication adherence in adults with chronic diseases: a systematic review and meta-analysis. International journal of epidemiology, 46(2), 589–602. https://doi.org/10.1093/ije/dyw273
